# Interesting Points from Half-Yearly Meeting

**Published:** 1918-07-27

**Authors:** 


### THE LEICESTER ROYAL INFIRMARY.

### Interesting Points from the Half-yearly Meeting.

THIS County Infirmary, its history and development during the last ten to fifteen years especially, is one of the most interesting studies anyone who follows the work and development of our voluntary hospitals could discover. Its administration has been characterised by great robust fearlessness, downright energetic efficiency, and the will to do and accomplish, with commendable results. Mr. T. Fielding Johnson, Junior, High Sheriff, presided at the half-yearly meeting held last week. Putting the patients first as the most important feature in the voluntary hospital, and the first cared for, a remarkable fact becomes apparent. The number of military in-patients fell from 531 to 186, whilst the civil cases flocked in and seized the opportunity of vacant beds with a zeal which increased the civil in-patient cases from 1,746 to 1,930 for the half-year. May we take this as an intimation of what to expect as an after-war result when the only peace the Allies can accept has been attained and consummated? Another remarkable fact is that the out-patients increased to 14,577, being 50 per cent. more than the corresponding half-year. Does this mean good or bad trade, or an epidemic of influenza, or what?

### A Vigorously Administered Voluntary Hospital.

At Leicester, as at many other vigorously administered voluntary hospitals, the orthopædic department in connection with that of the out-patients has outgrown its accommodation, due to the demands for treatment of discharged soldiers, which has resulted in Dr. Astley Clarke securing its removal to the theatre block attached to the children's hospital, where 350 cases a week are received. The department is in charge of a sister, assisted by four or five nurses who are undergoing teaching for massage certificates. It is typical of Leicester, and creditable to its board of management, that the latter has promptly provided the money to build a new in-patient department with an equipment of fifty beds, whilst the Mayor of Leicester, not to be outdone by any mere committee, has agreed to supply an out-patient department at a cost of £8,000. The board then took another hand, and decided to purchase property contiguous to the infirmary as a site for the Mayor's division, the cost of which amounts to several thousand pounds. It is not only not surprising, but just, that the refusal by the Ministry of Munitions to grant the necessary building permit should create a great deal of hostile comment and feeling, we understand, locally. Leicester, except its M.P. (one of the enemy group), deserves more considerate and just treatment having regard to the splendid work the city and county of Leicester have put in in helping to provide for wounded warriors, and to promote in every way the success of the Allies in the present awful war.

### Some Interesting Facts.

Mr. John Hodge, M.P., the Pensions Minister, is due to visit Leicester shortly, and the electorate are placing their faith in him to put the fear of God into those who are responsible for withdrawing the urgently needed accommodation for treatment by the working men of the city of Leicester. The Leicester Infirmary is ultimately to benefit to the extent of £10,000 from the estate of the late Sir Edward Wood, ex-chairman and life-long friend, whose memory it is proposed to perpetuate by the erection of a new wing, half the sum required being already given. This wing will add an extra thirty beds to the accommodation. The expenditure for the half-year has increased from upwards of £19,000 to upwards of £22,000, a result accounted for by an increase of £900 in the cost of provisions, of £960 for surgery and dispensary requisites, and of £700 for domestic expenses, all owing mainly to the advance in prices. Salaries and wages, too, demanded £900 more to foot the bill. Thanks to the great and successful effort of Mr. Alfred Corah, subscriptions yield an increase of £1,500. For some years, owing to the activity and progress so typical of Leicester, the business aptitude of its citizens, and the continuous increase in its resources, the city is finding, like other centres, that the demands upon and resulting work of the infirmary by its constant growth has caused the total cost of upkeep and maintenance to keep constantly ahead of the ever-growing funds.

### Privileges for the 500-Pounders.

The report mentioned the proposed Ministry of Health more than once, from the establishment of which its compilers seem to hope for the solution of many of the infirmary's difficulties. We shall hope to have an account of the method from which this happy result is forecast not later than the publication of the next half-yearly report. We congratulate those responsible for the administration of the Leicester Infirmary upon its decision to apply for the grant of a Royal Charter of Incorporation. Bodies of workpeople contributing to the Saturday Hospital Society not less than £500 during the five years ending December 31, 1917, are to possess the privilege of nominating a representative for election to a life governorship. This privilege will be extended to each subsequent contribution of £500, provided that no one body of workpeople shall nominate for election under this new rule more than three representatives.

